# BioInnovate AI: A Machine Learning Platform for Rapid PCR Assay Design in Emerging Infectious Disease Diagnostics

**DOI:** 10.3390/diagnostics15121445

**Published:** 2025-06-06

**Authors:** Hung-Hsin Lin, Hsing-Yi Chung, Tai-Han Lin, Chih-Kai Chang, Cherng-Lih Perng, Kuo-Sheng Hung, Katsunori Yanagihara, Hung-Sheng Shang, Ming-Jr Jian

**Affiliations:** 1 Division of Clinical Pathology, Department of Pathology, Tri-Service General Hospital, National Defense Medical Center, Taipei 114, Taiwan; 2 Center for Precision Medicine and Genomics, Tri-Service General Hospital, National Defense Medical Center, Taipei 114, Taiwan; 3 Department of Laboratory Medicine, Nagasaki University Graduate School of Biomedical Sciences, Nagasaki 852-8501, Japan

**Keywords:** emerging infectious diseases, artificial intelligence, PCR design, medical diagnostics

## Abstract

**Background/Objectives:** Emerging infectious diseases pose significant global threats due to their rapid transmission, limited therapeutic options, and profound socioeconomic impact. Conventional diagnostic techniques that rely on sequencing and polymerase chain reactions (PCR) frequently lack the speed necessary to efficiently respond to rapidly evolving pathogens. This study describes the development of BioInnovate AI to overcome these limitations using machine learning to expedite PCR assay development. **Methods**: The ability of BioInnovate AI to predict optimal PCR reagents across multiple pathogens was assessed. Additionally, random forest classifier, light gradient boosting machine (LGBM), and gradient boosting classifier models were evaluated for their ability to predict effective PCR primer–probe combinations. Performance metrics, including the area under the curve (AUC), sensitivity, specificity, accuracy, and F1 score, were assessed to identify the optimal model for platform integration. **Results**: All machine learning models performed well, with the LGBM model achieving the highest metrics (AUC: 0.97, sensitivity: 0.93, specificity: 0.91). BioInnovate AI significantly reduced PCR assay development time by approximately 90%, enabling rapid design and reagent optimization for multiple pathogens. **Conclusions**: BioInnovate AI provides a rapid, accurate, and efficient method for PCR reagent design, significantly enhancing global diagnostic preparedness by optimizing primers and probes for the timely detection of infectious diseases.

## 1. Introduction

The World Health Organization (WHO) has highlighted the critical global health security challenges posed by emerging infectious diseases (EIDs) characterized by high transmission risks, limited treatment options, and profound socioeconomic disruptions [1,2]. The coronavirus disease (COVID-19) pandemic, resulting in over six million deaths and an estimated economic loss exceeding $17 trillion, serves as a stark reminder of the potentially devastating impact of EIDs [3,4,5,6,7]. Similar examples include the Ebola virus, with fatality rates approaching 90% in certain regions [8], and the H1N1 influenza pandemic, responsible for 151,700–575,400 deaths worldwide within its first year [9], underscoring the urgent need for robust diagnostic capabilities.

Traditional diagnostic processes for EIDs typically begin with sequencing methods, such as Sanger sequencing, a precise but time-consuming process that can take days to weeks to produce results [10,11]. After sequencing, polymerase chain reaction (PCR) primer and probe design and validation are required, further delaying the development of effective diagnostic tools [10,11]. Despite being reliable, PCR often lacks the speed and adaptability to quickly respond to novel and rapidly mutating pathogens [10,11]. The rising frequency and severity of EID outbreaks necessitate faster, more adaptable diagnostic approaches [12].

Emerging pathogens like influenza viruses and coronaviruses can frequently mutate, rendering the existing diagnostic reagents ineffective [12,13]. This bottleneck can hamper the detection and management of infectious diseases, compromising public health efforts. The primary challenge is the lengthy process of designing and validating PCR primers and probes [2]. Traditional methods are not equipped to handle the urgent need for rapid reagent development [14,15]. Thus, an innovative solution that leverages artificial intelligence and machine learning is crucial to streamline and optimize PCR primer and probe design.

This research aims to design a novel “BioInnovate AI” platform, employing machine learning algorithms to predict the likelihood of successful amplification using current primers to detect emerging pathogens and less-studied targets, such as invasive species. BioInnovate AI effectively streamlines the design process for complex targets, with considerable versatility in supporting diverse molecular diagnostic applications. This innovation ensures more rapid and accurate detection, reduces the time and resources required for reagent development, and augments the capacity of laboratories to swiftly address infectious diseases and ecological threats.

## 2. Materials and Methods

### 2.1. Assay Development

Bacterial and viral gene sequences were retrieved from the NCBI database using “Homo sapiens” classification, complete assembly levels, and a focused timeframe of 2023–2024. Sequences exhibiting atypical assemblies were removed, and the remaining were downloaded in FASTA format. Using the Primer Express software 3.0.1, oligonucleotide primers (forward and reverse) were designed with attention to sequence characteristics, lengths, and melting temperatures, as outlined in Tables S1 and S2 for SYBR and TaqMan, respectively. The alignment process involved feature extraction, including mismatch identification, melting temperature assessment, and end-mismatch ratio calculations, to ensure optimal primer–target compatibility. The quantitative polymerase chain reaction (qPCR) assays evaluated the specificity of 14 respiratory pathogens [16], categorized into viral and bacterial groups. The viral group includes adenovirus, human metapneumovirus, human parainfluenza virus (types 1, 2, and 3), enteroviruses, respiratory syncytial virus, and influenza virus. The bacterial group comprised *Chlamydia*, *Haemophilus influenzae*, *Streptococcus pneumoniae*, *Legionella pneumophila*, *Mycoplasma pneumoniae*, and *Staphylococcus aureus.* Ten invasive species were incorporated to enhance genomic data comprehensiveness, ensuring a comprehensive analytical approach [17].

**Figure 1:**
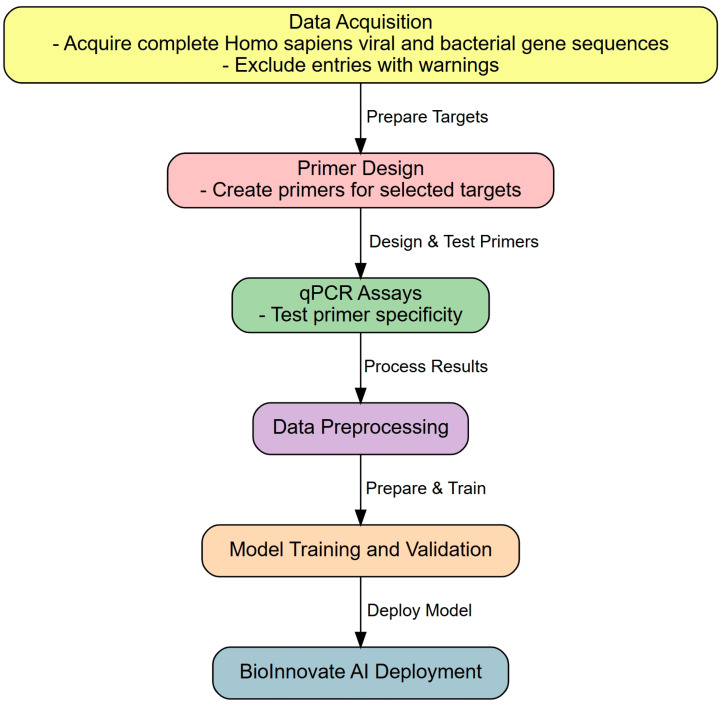
Workflow of the BioInnovate AI platform. Starting with gene sequence retrieval from NCBI, the BioInnovate AI process involves primer and probe design using the Primer Express software. Features such as mismatches and melting temperatures are extracted to train the machine learning models. These trained models are integrated into a user-friendly web platform (“BioInnovate AI”), enabling sequence input, primer optimization, and amplification success prediction to efficiently streamline assay development.

SYBR Green and TaqMan were used for qPCR assays. The thermal cycling conditions included an initial denaturation at 95 °C for 10 min, followed by 45 cycles of 95 °C for 15 s and 60 °C for 1 min. Fluorescence thresholds were set at 0.2 ΔRn to ensure consistency across replicates, with amplification deemed positive if it occurred in any of the triplicate wells. This integrated workflow, from NCBI data retrieval to qPCR assay validation, provided a robust methodological foundation for addressing the diagnostic challenges posed by respiratory pathogens and invasive species (Figure 1).

### 2.2. Training Data Pre-Processing

The training dataset was constructed by integrating the specificity test results with comprehensive mismatch information from the assay templates to identify the critical variables impacting PCR assay performance. These elements were selected to construct a robust model capable of predicting assay performance with high accuracy.

Primer analysis was based on total mismatch counts across primer sets (total_mm), average length (avg_length), mean melting temperature of primers (Tm_mean), temperature differences between primer pairs (Tm_diff), and absolute mismatch difference normalized by total_mm (mm_abs_diff). Additionally, the proportion of mismatches was evaluated for the first five base pairs of the 3′ primer ends (3p_mm_percent) and the 3′ primer termini (term_mm_percent). To provide detailed insights into the effects of genetic variation, the total mismatch counts across primer sets were categorized into four nucleotide change types: transitions involving purine-to-purine or pyrimidine-to-pyrimidine changes (AB_mm_percent), transversions involving purine-to-pyrimidine changes (TV_mm_percent), cytosine-to-cytosine mismatches (CC_mm_percent), and guanine-to-guanine mismatches (GG_mm_percent). These proportional metrics enabled analysis of how different nucleotide substitution patterns influence PCR amplification efficiency.

TaqMan assays were examined, with probe-specific attributes quantified by analyzing the total mismatches (P_total_mm), probe length (P_length), and melting temperature (P_Tm), with particular focus on central sequence variations (P_mm_center) representing mismatches outside the first five base pairs at either end. Probe mismatch analysis was further refined by categorizing nucleotide changes into P_AB_mm_percent, P_TV_mm_percent, P_CC_mm_percent, and P_GG_mm_percent, providing a framework for understanding factors influencing PCR performance.

The dataset incorporated all calculated features, with percentage-based metrics representing 0% in the absence of mismatches. This ensured complete data representation, with no missing values requiring imputation. All features used for model training were continuous variables derived from quantitative calculations; only outcome variables were categorical, indicating successful PCR amplification (1) or failure (0). This provided a robust and observable foundation for model development and validation.

### 2.3. Model Training, Validation, and Metrics Evaluation

The model was developed using Python 3.9.18 and a scikit-learn framework. The SYBR Green assay results revealed 779 positive and 653 negative samples, whereas the TaqMan assay showed 858 positive and 791 negative samples. Various predictive models—random forest classifier (RFC), light gradient boosting machine (LGBM), and gradient boosting classifier (GBC)—were integrated into the methodology for the optimization and training phases. The dataset was organized into distinct features and binary outcomes, delineating the presence or absence of amplification to ensure precise model predictions. To validate the models, the dataset was randomly partitioned in a 7:3 ratio for training and validation, enhancing the reliability and generalizability of the analytical approach.

A 10-fold cross-validation across 10 cycles was performed, with a consistent random state to ensure reproducibility of the results across all models. A grid search was also performed to identify the optimal parameters for each predictive model (Table S3).

Model interpretability was improved using random forest feature importance assessment and Shapley additive explanations (SHAP) analysis, providing global and local insights into feature contributions. Features with zero importance values were excluded from the final model to reduce noise. Heat maps were generated to visualize the relationships between key features and PCR amplification outcomes, identifying the most influential variables in determining assay success.

Model efficacy was evaluated based on sensitivity, specificity, positive predictive value (PPV), negative predictive value (NPV), F1 score, accuracy, and area under the curve (AUC). These metrics provide insights into model accuracy, precision–recall balance, and diagnostic capabilities.

### 2.4. Development of a User Interface

A user interface was developed using the Flask Web framework and Python 3.9.18 to facilitate interactions with the predictive models. The Flask framework was selected for its simplicity and efficiency, enabling the creation of accessible platforms to employ the trained models.

The interface design process began with constructing a web form to accept user inputs, including genetic sequences in the FASTA format, forward and reverse primers, and their associated melting temperatures (Tm). The Pandas library was utilized for data handling, BioPython’s SeqIO 1.83 for parsing the sequence data, and Joblib 1.3.2 to integrate the pretrained machine learning models into the application.

## 3. Results

### 3.1. Key Influencers in Amplification Success

**Figure 2:**
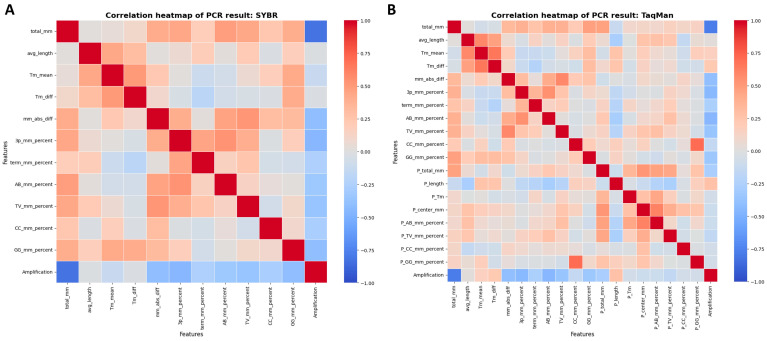
Correlation heatmaps of molecular features influencing PCR amplification success. Heatmaps depicting the relationships between key features and amplification outcomes for the SYBR Green (**A**) and TaqMan (**B**) assays. An increase in total mismatches negatively correlates with amplification success in both methodologies.

Within the SYBR Green and TaqMan datasets, amplification success consistently correlated negatively with mismatch-related features, such as total_mm, 3p_mm_percent, term_mm_percent, and various nucleotide change types (Figure 2A,B). Hence, increased mismatches directly reduced the likelihood of successful PCR amplification using both assay methods. The feature importance analysis revealed consistent patterns, with the top five key influencers for SYBR Green being total_mm, 3p_mm_percent, AB_mm_percent, TV_mm_percent, and mm_abs_diff, while the TaqMan dataset highlighted total_mm, 3p_mm_percent, AB_mm_percent, TV_mm_percent, and P_total_mm.

To further elucidate the influence of each feature on model predictions, SHAP summary plots were generated, displaying the top ten features ranked by importance (Figure S1A,B). In both datasets, total primer and 3′ end mismatches were consistently among the most influential predictors, with higher values generally associated with a lower likelihood of successful amplification.

### 3.2. Model Validation and Performance Metrics Overview

**Figure 3:**
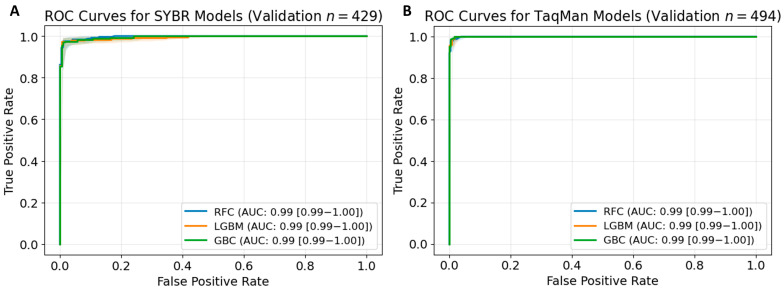
Receiver operating characteristic (ROC) curves for predicting PCR amplification success using random forest classifier (RFC), light gradient boosting machine (LGBM), and gradient boosting classifier (GBC) models. Validation sets for (**A**) SYBR (*n* = 429) and (**B**) TaqMan (*n* = 494). The area under the curve (AUC) values and 95% confidence intervals (CI) for each model are presented; shaded regions represent the 95% CI for the ROC curves.

All predictive models demonstrated strong predictive capabilities (Table 1), achieving AUC values of 0.99 across both the training and validation datasets for SYBR Green (Figure 3A) and TaqMan (Figure 3B). In the SYBR Green dataset, the RFC demonstrated the highest performance, with sensitivity, specificity, and accuracy scores reaching or exceeding 0.97 and with an AUC of 0.99. The LGBM and GBC achieved strong metrics, with sensitivities and accuracy at 0.95 and 0.97, respectively. However, GBC achieved a slightly higher specificity (0.99) than LGBM (0.98). Nevertheless, both models displayed reliable results. For the TaqMan dataset, LGBM achieved the highest sensitivity (0.99), accuracy (0.99), and F1 score (0.99). RFC followed closely with metrics of 0.98–0.99 across all evaluation parameters. Although GBC achieved a slightly lower F1 score (0.97), it maintained a sensitivity and specificity of 0.98. Given its overall reliability, RFC was selected as the core model for the SYBR Green and TaqMan datasets on the BioInnovate AI platform.

### 3.3. Development of User Interface

**Figure 4:**
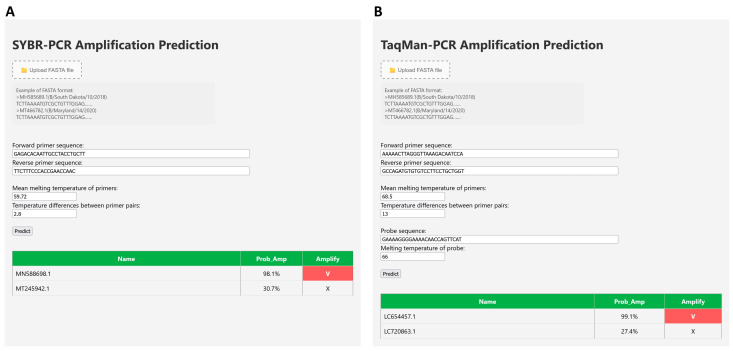
User interface for amplification prediction in the BioInnovate AI platform. (**A**) The SYBR-PCR amplification prediction interface allows users to upload FASTA files, enter forward and reverse primer sequences, and provide primer melting temperature information and temperature differences. (**B**) The TaqMan-PCR interface extends this functionality by enabling probe sequence and probe melting temperature input. Both interfaces display predicted amplification probabilities in a results table, with successful amplification indicated by a red “✔” and failure by a gray “✘”.

The user interfaces for the SYBR PCR (Figure 4A) and TaqMan PCR (Figure 4B) amplification prediction tools within the BioInnovate AI platform provide examples of the required FASTA file format. Users input primer and probe sequences with their Tm parameters to obtain the probability of amplification success within seconds. The tools generate a summary table displaying the amplification probabilities and highlight successful amplifications with a red background, offering a clear visual cue for users.

### 3.4. Practical Implementation and Impact on PCR Assay Design

Overall, mismatch-related features emerged as critical determinants of PCR amplification success. The strong performance of the RFC model across SYBR Green and TaqMan assays, combined with an intuitive web-based platform, provides a reliable, data-driven framework for optimizing primer and probe designs. Researchers can iteratively refine primer and probe sequences by systematically adjusting nucleotides and leveraging the model’s predictive capabilities to optimize the likelihood of successful amplification.

## 4. Discussion

This research introduces the BioInnovate AI platform, which significantly enhances the design and optimization of PCR reagents through advanced machine-learning algorithms. This platform predicts the likelihood of successful amplification using existing primers for specified targets, thereby decreasing the time and resources required for PCR reagent development. All model evaluations exhibited performance metrics exceeding 0.9, ensuring highly reliable and accurate predictions. This innovation improves the speed and accuracy of pathogen detection, enabling laboratories to respond swiftly to emerging infectious threats.

This study validated machine learning models using multiple metrics to evaluate their predictive capabilities. SYBR models had AUC values of 0.99 for training and validation datasets, demonstrating consistent performance. The RFC achieved the highest sensitivity (0.97), specificity (0.99), PPV (0.99), NPV (0.97), F1 score (0.98), and accuracy (0.98) among the SYBR models. For TaqMan assays, RFC and LGBM achieved AUC values of 0.99. LGBM showed a sensitivity of 0.99, F1 score of 0.99, and accuracy of 0.99, whereas RFC exhibited a specificity of 0.99 and PPV of 0.99. These results indicate reliable predictions with balanced diagnostic precision and recall.

EIDs have significantly impacted mortality and financial stability worldwide [18]. Historical examples include the Black Death and the 1918 influenza pandemic, with death tolls of 75–200 million and ~50 million, respectively [3,8,9]. Meanwhile, the COVID-19 pandemic has underscored the urgent need for rapid and reliable diagnostic tools. Innovations like BioInnovate AI can markedly shorten detection and response times, potentially saving lives and reducing economic losses.

Primer3 and Primer-BLAST are widely employed to design PCR primers using rule-based algorithms and database searches [19,20]. These tools generate candidate primers based on sequence characteristics and customizable parameters such as length, melting temperature, and GC content. NCBI Primer-BLAST further incorporates specificity checks to prevent off-target amplification and accommodates genomic features such as exon junctions or single nucleotide polymorphisms [19,20]. Despite their strengths in primer generation and initial screening, these tools do not predict amplification success under experimental conditions or adapt based on empirical results. Similarly, although machine learning approaches like eDNAssays have improved specificity prediction, they focus primarily on environmental DNA applications rather than clinical diagnostics [17]. BioInnovate AI addresses these limitations by integrating empirical qPCR data with key thermodynamic parameters within an ensemble machine learning framework, enabling precise prediction of amplification success and streamlining workflows through a user-friendly interface. This platform supports rapid primer optimization for pathogen detection, potentially reducing the development time and resource expenditure by up to 90%, making BioInnovate AI a choice for diagnostic assay development.

PCR involves a heat–denature–annealing cycle utilizing a heat-tolerant polymerase to amplify specific DNA sequences [21]. Viruses and bacteria have unique genetic sequences that can be identified by PCR [21]. Therefore, primer design is crucial [22], as it ensures the specificity and sensitivity of a PCR assay. However, conventional primer design can take over two weeks [22], extended by reagent synthesis at biotechnology companies, such as Integrated DNA Technologies, Inc. (IDT) [23], and validation procedures. BioInnovate AI designs primers and predicts the likelihood of amplification within minutes, reducing the time required and transforming public health response capabilities with rapid, precise diagnostic tools for diverse pathogens.

BioInnovate AI has also been developed with clinical implementation in mind. Its scalable architecture integrates electronic health records and laboratory systems, ensuring efficient real-time information exchange. Moreover, its flexible machine learning framework can be adapted to address various diagnostic challenges. Integrating computational analytics with molecular diagnostics underscores the role of interdisciplinary collaboration in advancing precision medicine and improving clinical outcomes [24,25].

Despite its potential, the BioInnovate AI platform has some limitations. Model accuracy depends on the training data quality and comprehensiveness, with potential biases affecting performance. Although the study dataset incorporated diverse respiratory pathogens from the NCBI for the Biotechnology Information database, underrepresented variants in public databases may introduce selection bias. Moreover, implementing the platform in resource-limited settings may be challenging due to the need for computational infrastructure and machine-learning expertise. Additionally, this feature engineering approach focuses on mismatch and thermodynamic variables, potentially missing other biochemical factors such as secondary structure formation or local sequence context effects. Finally, the high AUC values in the SYBR Green and TaqMan models, while indicating strong discriminative power, may suggest overfitting despite rigorous cross-validation procedures. Therefore, the platform predictions require further validation across diverse real-world settings and independent laboratories to ensure reliability, generalizability, and clinical utility.

Future research could improve the robustness of platforms such as BioInnovate AI by expanding training datasets to include a wider variety of clinically relevant and emerging pathogens, while validation across independent multicenter datasets would strengthen generalizability. Incorporating additional sequence-derived features and experimental metadata would better capture PCR dynamics. The principles outlined in this study could also be extended to multiplex PCR design and CRISPR-based diagnostics with tailored modeling approaches. Moreover, collaborative validation across multiple laboratories would provide valuable insights into real-world performance variations and accelerate the adoption of AI-driven tools in infectious disease diagnostics.

## 5. Conclusions

The novel BioInnovate AI platform considerably reduces the PCR reagent development time by 90%, enabling the rapid detection of diverse pathogens. This innovation strengthens global diagnostic capacity, supporting timely surveillance, clinical interventions, and improved outcomes for EIDs.

## Figures and Tables

**Table 1:** Detailed metrics for trained models.

	Training AUC	Validating AUC	Sensitivity	Specificity	PPV	NPV	F1 Score	Accuracy
SYBR models								
RFC	0.99	0.99	0.97	0.99	0.99	0.97	0.98	0.98
LGBM	0.99	0.99	0.95	0.98	0.99	0.95	0.97	0.97
GBC	0.99	0.99	0.95	0.99	0.99	0.95	0.97	0.97
TaqMan models								
RFC	0.99	0.99	0.98	0.99	0.99	0.98	0.99	0.99
LGBM	0.99	0.99	0.99	0.98	0.98	0.99	0.99	0.99
GBC	0.99	0.99	0.98	0.98	0.98	0.97	0.98	0.98

Abbreviations: RFC, random forest classifier; LGBM, light gradient boosting machine; GBC, gradient boosting classifier; PPV, positive predictive value; NPV, negative predictive value; AUC, area under the curve.

## Data Availability

The data is available from the corresponding author upon reasonable request.

## Supplementary Materials

The following supporting information can be downloaded at: https://www.mdpi.com/article/10.3390/diagnostics15121445/s1. Table S1: SYBR Green Parameters; Table S2: TaqMan Chemistry Parameters. Table S3: Grid Search Parameters of Machine Learning Models; Figure S1: Top 10 SHAP Features for Amplification Prediction for Both Models.

## Abbreviations

The following abbreviations are used in this manuscript:

PCR	Polymerase Chain Reaction
qPCR	quantitative Polymerase Chain Reaction
EID	Emerging Infectious Disease
Tm	Melting Temperature
RFC	Random Forest Classifier
LGBM	Light Gradient Boosting Machine
GBC	Gradient Boosting Classifier
AUC	Area Under the Curve
PPV	Positive Predictive Value
NPV	Negative Predictive Value
ROC	Receiver Operating Characteristic
