# Supplementary material for: BioInnovate AI: A Machine Learning Platform for Rapid PCR Assay Design in Emerging Infectious Disease Diagnostics
Source: Diagnostics (Basel). 2025 Jun 6;15(12):1445. doi: 10.3390/diagnostics15121445 (PMC12191490; doi:10.3390/diagnostics15121445)
Supplement: Supplementary file 1 [file diagnostics-15-01445-s001.zip › Supplementary Figure S1.pdf]

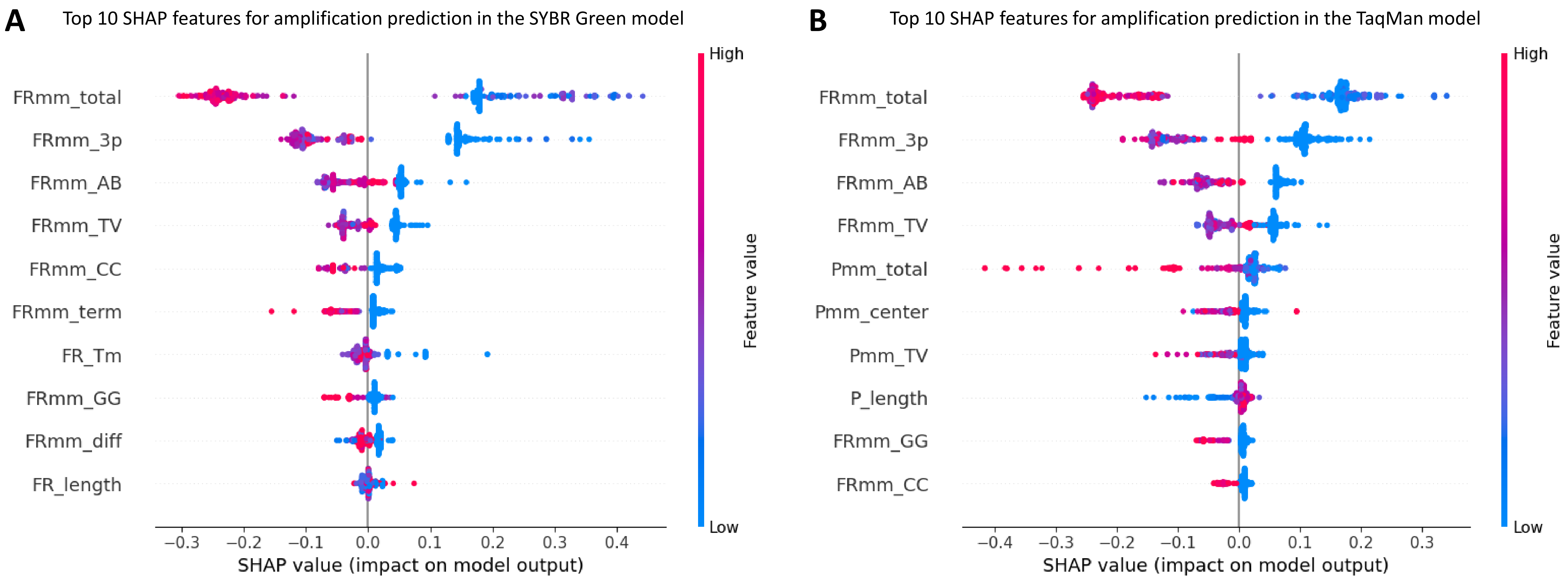
A
Top 10 SHAP features for amplification prediction in the SYBR Green model
FRmm_total
FRmm_3p
FRmm_AB
FRmm_TV
FRmm_CC
FRmm_term
FR_Tm
FRmm_GG
FRmm_diff
FR_length
−0.3 −0.2 −0.1 0.0 0.1 0.2 0.3 0.4
SHAP value (impact on model output)
High
Low
Feature value
B
Top 10 SHAP features for amplification prediction in the TaqMan model
FRmm_total
FRmm_3p
FRmm_AB
FRmm_TV
Pmm_total
Pmm_center
Pmm_TV
P_length
FRmm_GG
FRmm_CC
−0.4 −0.3 −0.2 −0.1 0.0 0.1 0.2 0.3
SHAP value (impact on model output)
High
Low
Feature value
