# Supplementary material for: BioInnovate AI: A Machine Learning Platform for Rapid PCR Assay Design in Emerging Infectious Disease Diagnostics
Source: Diagnostics (Basel). 2025 Jun 6;15(12):1445. doi: 10.3390/diagnostics15121445 (PMC12191490; doi:10.3390/diagnostics15121445)
Supplement: Supplementary file 1 [file diagnostics-15-01445-s001.zip › Supplementary Tables.pdf]

Supplementary Table S1. SYBR Green Parameters

**Pathogen Classification**	**Forward Primer**		**Reverse Primer**		**PCR reaction**	
	Sequence	*T*m (°C)	Sequence	*T*m (°C)	No. detected	No. non-detected
**Virus**						
**Adenovirus**	GCCCCAGTGGTCTTACATGCACATC<br>Sequence ID: OR777209.1	72	GCCACGGTGGGGTTTCTAAACTTGTT<br>Sequence ID: PQ336882.1	72	11	0
		72	CGCACGGTGGGTTTCTTAAACTCGTT<br>Sequence ID: XM_073708295.1	72	0	11
**Human metapneumovirus**	CAAGTGTGACATTGCTGACCTGAA<br>Sequence ID: KU821121.1	67	ACTGCCGCACAACATTTAGAAA<br>Sequence ID: PQ634903.1	65	20	0
		67	GCTGCGACATAAGTTTACCAAA<br>Sequence ID: XM_051813484.1	65	0	20
**Human Parainfluenza Virus Type 1**	AAAAACTTAGGGTTAAAGACAATCCA<br>Sequence ID: PP757765.1	62	GCCAGATGTGTGTCCTTCCTGCTGGT<br>Sequence ID: PP886694.1	75	100	0
		62	TGCAGCTGTCTGGCCTGCTTGGCTGT<br>Sequence ID: CP073346.1	75	2	98
**Human Parainfluenza Virus Type 2**	CCATTTACCTAAGTGATGGAA<br>Sequence ID: PV260512.1	59	CGTGGCATAATCTTCTTTTT<br>Sequence ID: PV224521.1	58	49	0
		59	CTGCGCATAATCTATTCTTT<br>Sequence ID: AP038838.1	58	0	49
		59	TGCCGATAATCTTTTCTTTT<br>Sequence ID: CP117839.1	58	0	49
**Human Parainfluenza Virus Type 3**	CAGGAAGCATTGTATCATCTGT<br>Sequence ID: OQ981731.1	62	ATAGTGTGTAATGCAGCTTGT<br>Sequence ID: OQ981759.1	61	49	0
		62	ATGTTGTGAAATGCAGTCTTG<br>Sequence ID: CP072844.1	61	0	49
**Enterovirus**	CCCCTGAATGCGGCTAATC<br>Sequence ID: PP838728.1	60.4	GATTGTCACCATAAGCAGC<br>Sequence ID: PP548279.1	50.7	30	30

**Respiratory syncytial virus**	CTGTCATCCAGCAAATACAC<br>Sequence ID: PV367374.1	59.6	GCATATAACATACCTATTAAACC<br>Sequence ID: PV367290.1	55.9	20	0
**Influenza virus**	GAGACACAATTGCCTACCTGCTT<br>Sequence ID: MK380264.1	61.1	TTCTTTCCCACCGAACCAAC<br>Sequence ID: MK857775.1	58.3	88	10
		61.1	CCACTTACCGTCAACTCCAT<br>Sequence ID: CP119764.1	58.3	0	98
**Bacteria**						
Chlamydia	CGTTGGTTTATTCGGAGTTA<br>Sequence ID: LN846995.1	48	CCAAGAGAAAGAGGTGTCTGT<br>Sequence ID: LN847227.1	52	9	0
		48	GGAACTGAAGCTGAGTTCTGT<br>Sequence ID: CP185739.1	52	9	0
Haemophilus influenzae	CACCATCGGCATATTTAACCACT<br>Sequence ID: CP095962.1	63	AGATTGGAAAGAAACACAAGAAAAAGA<br>Sequence ID: CP095996.1	63	30	0
		63	AAGTTGGAAGAAACACAAGAATAAAGA<br>Sequence ID: CP185739.1	63	0	30
Streptococcus pneumoniae	TAAACAGTTTGCCTGTAGTCG<br>Sequence ID: CP118286.1	63	CCCGGATATCTCTTTCTGGA<br>Sequence ID: CP137102.1	63	17	0
		63	GGCCGATCTTCTTATCTCGA<br>Sequence ID: CP102405.1	63	0	17
Legionella pneumophila	GGCTTTAACCGAACAGCAAA<br>Sequence ID: AP038846.1	63	TTGCAAACCACTTGGCAATA<br>Sequence ID: AP038846.1	63	49	0
		63	TGGCAAACCATTGGACACTA<br>Sequence ID: in this study	63	46	3
		63	TGGCAAACGATTGGTCACAA<br>Sequence ID: CP041767.1	63	0	49
Mycoplasma pneumoniae	TGTACCAGAGCACCCCAGAAG<br>Sequence ID: CP017340.1	53	GGTCGGCACGAATTTCATATAAG<br>Sequence ID: CP010544.1	54	62	0

		53	GGTAGGCATGAATTCCATGTAAG<br>Sequence ID: CP033459.1	54	62	0
Staphylococcus aureus	GCCTTATTCGAAGAAACCG<br>Sequence ID: CP162456.1	56	CTACTCTTCTGAAAAGCGTCG<br>Sequence ID: CP123149.1	62	2	2

Supplementary Table S2. TaqMan Chemistry Parameters

Pathogen Classification	Forward Primer		Reverse Primer		Probe		PCR reaction	
	Sequence	*T*m (°C)	Sequence	*T*m (°C)	Sequence	*T*m (°C)	No. detected	No. non-detected
**Virus**								
**Adenovirus**	GCCCCAGTGGTCTTACATGCACATC<br>Sequence ID: OR777209.1	72	GCCACGGTGGGGTTTCTAAACTTGTT<br>Sequence ID: PQ336882.1	72	TCGGAGTACCTGAGCCCGGGTCTGGTGCA<br>Sequence ID: AB679751.1	68.6	11	0
		72	CGCACGGTGGGTTTCTTAAACTCGTT<br>Sequence ID: XM_073708295.1	72	TCGGAGTACCTGAGCCCGGGTCTGGTGCA<br>Sequence ID: AB679751.1	68.6	0	11
**Human metapneumovirus**	CAAGTGTGACATTGCTGACCTGAA<br>Sequence ID: KU821121.1	67	ACTGCCGCACAACATTTAGAAA<br>Sequence ID: PQ634903.1	65	TGGCCGTTAGCTTCAGTCAATTCAACAGA<br>Sequence ID: KC562243.1	72	15	5
		67	GCTGCGACATAAGTTTACCAAA<br>Sequence ID: XM_051813484.1	65	TGGCCGTTAGCTTCAGTCAATTCAACAGA<br>Sequence ID: KC562243.1	72	0	20
**Human Parainfluenza Virus Type 1**	AAAAACTTAGGGTTAAAGACAATCCA<br>Sequence ID: PP757765.1	62	GCCAGATGTGTGTCCTTCCTGCTGGT<br>Sequence ID: PP886694.1	75	GAAAAGGGGAAAACAACCAGTTCAT<br>Sequence ID: PP886689.1	66	84	16
		62	GCCAGATGTGTGTCCTTCCTGCTGGT<br>Sequence ID: PP886694.1	75	GCCAACCCACAAGGCAACAACATCT<br>Sequence ID: PP757765.1	73	94	6
		62	GCCAGATGTGTGTCCTTCCTGCTGGT<br>Sequence ID: PP886694.1	75	GGTCTACAACCCGAAATGATAACTC<br>Sequence ID: PP886690.1	65	100	0
		62	TGCAGCTGTCTGGCCTGCTTGGCTGT<br>Sequence ID: CP073346.1	75	GGTCTACAACCCGAAATGATAACTC<br>Sequence ID: PP886690.1	66	0	100
**Human Parainfluenza Virus Type 2**	CCATTTACCTAAGTGATGGAA<br>Sequence ID: PV260512.1	59	CGTGGCATAATCTTCTTTTT<br>Sequence ID: PV224521.1	58	AATCGCAAAAGCTGTTCAGTCAC<br>Sequence ID: MN369034.1	66	49	0
		59	CGTGGCATAATCTTCTTTTT<br>Sequence ID: PV224521.1	58	GCTATACCAGGAGGCTGTGTCTTAT<br>Sequence ID: PV498569.1	76	32	17
		59	CGTGGCATAATCTTCTTTTT<br>Sequence ID: PV224521.1	58	AGGAGGCTGTGTCTTATATTGCTAT<br>Sequence ID: PV260512.1	76	32	17

		59	CTGCGCATAATCTATTCTTT<br>Sequence ID: AP038838.1	58	AATCGCAAAAGCTGTTCAGTCAC<br>Sequence ID: OP672261.1	66	0	49
		59	TGCCGATAATCTTTTCTTTT<br>Sequence ID: CP117839.1	58	AATCGCAAAAGCTGTTCAGTCAC<br>Sequence ID: OP672261.1	66	0	49
**Human Parainfluenza Virus Type 3**	CAGGAAGCATTGTATCATCTGT<br>Sequence ID: OQ981731.1	62	ATAGTGTGTAATGCAGCTTGT<br>Sequence ID: OQ981759.1	61	ACCCAGTCATAACTTACTCAACAGCAAC<br>Sequence ID: OQ981759.1	68	49	0
		62	ATGTTGTGAAATGCAGTCTTG<br>Sequence ID: CP072844.1	61	ACCCAGTCATAACTTACTCAACAGCAAC<br>Sequence ID: OQ981759.1	68	0	49
**Enterovirus**	CCCCTGAATGCGGCTAATC<br>Sequence ID: PP838728.1	60.4	GATTGTCACCATAAGCAGC<br>Sequence ID: PP548279.1	50.7	CGGAACCGACTACTTTGGGTGTCCGT<br>Sequence ID: PP838728.1	72.7	29	31
**Respiratory syncytial virus**	CTGTCATCCAGCAAATACAC<br>Sequence ID: PV367374.1	59.6	GCATATAACATACCTATTAAACC<br>Sequence ID: PV367290.1	55.9	GTGCAGAAACACATTAATAAGTTAT<br>Sequence ID: PV080940.1	59.6	11	9
		59.6		55.9	TAAGTTATGTGGTATGTTATTAATC<br>Sequence ID: PP784985.1	59.6	11	9
		59.6		55.9	GTTATTAATCACAGAAGATGCTAAT<br>Sequence ID: PQ066216.1	59.6	12	8
		59.6		55.9	GAGCACAGGAGACAGCATTGA<br>Sequence ID: PQ762756.1	59.6	14	6
**Influenza virus**	GAGACACAATTGCCTACCTGCTT<br>Sequence ID: MK380264.1	61.1	TTCTTTCCCACCGAACCAAC<br>Sequence ID: MK857775.1	58.3	AGAAGATGGAGAAGGCAAAGCAGAACTAGC<br>Sequence ID: MN819450.1	66.6	88	10
**Bacteria**								
Chlamydia	CGTTGGTTTATTCGGAGTTA<br>Sequence ID: LN846995.1	48	CCAAGAGAAAGAGGTGTCTGT<br>Sequence ID: LN847227.1	52	TGTAAATGCAAATGAACTACCAAACGTTTC<br>Sequence ID: LN846995.1	56	9	0
		48	GGAACTGAAGCTGAGTTCTGT<br>Sequence ID: CP185739.1	52	TGTAAATGCAAATGAACTACCAAACGTTTC<br>Sequence ID: LN846995.1	56	0	9
Haemophilus influenzae	CACCATCGGCATATTTAACCACT<br>Sequence ID: CP095962.1	63	AGATTGGAAAGAAACACAAGAAAAAGA<br>Sequence ID: CP095996.1	63	AAACATCCAATCGTAATTATAG<br>Sequence ID: CP095958.1	63	30	0

		63	AAGTTGGAAGAAACACAAGAATAAAGA<br>Sequence ID: CP185739.1	63	AAACATCCAATCGTAATTATAG<br>Sequence ID: CP095958.1	63	0	30
Streptococcus pneumoniae	TAAACAGTTTGCCTGTAGTCG<br>Sequence ID: CP118286.1	63	CCCGGATATCTCTTTCTGGA<br>Sequence ID: CP137102.1	63	AACCTTTGTTCTCTCTCGTGGCAGCTCAA<br>Sequence ID: CP137102.1	63	17	0
		63	GGCCGATCTTCTTATCTCGA<br>Sequence ID: CP102405.1	63	AACCTTTGTTCTCTCTCGTGGCAGCTCAA<br>Sequence ID: CP137102.1	63	0	17
Legionella pneumophila	GGCTTTAACCGAACAGCAAA<br>Sequence ID: AP038846.1	63	TTGCAAACCACTTGGCAATA<br>Sequence ID: CP010544.1	63	CAAAAACAAGCCAGGCGTTGTTG<br>Sequence ID: AP038846.1	63	49	0
		63	TGGCAAACCATTGGACACTA<br>Sequence ID: OZ255038.1	63	CAAAAACAAGCCAGGCGTTGTTG<br>Sequence ID: AP038846.1	63	0	49
Mycoplasma pneumoniae	TGTACCAGAGCACCCCAGAAG<br>Sequence ID: CP017340.1	53	GGTCGGCACGAATTTCATATAAG<br>Sequence ID: CP010544.1	54	TGTACCAGAGCACCCCAGAAGG<br>Sequence ID: LR214945.1	62.6	62	0
		53	GGTAGGCATGAATTCCATGTAAG<br>Sequence ID: CP033459.1	54	TGTACCAGAGCACCCCAGAAGG<br>Sequence ID: LR214945.1	62.6	0	62
Staphylococcus aureus	GCCTTATTCGAAGAAACCG<br>Sequence ID: CP162456.1	56	CTACTCTTCTGAAAAGCGTCG<br>Sequence ID: CP123149.1	62	ATGAGAGATATGCGAATATGAG<br>Sequence ID: CP162456.1	65	1	3

Supplementary Table S3. Grid Search Parameters of Machine Learning Models

**Model**	**Parameter**	**SYBR Green**	**TaqMan Chemistry**
**Random Forest Classifier**	n_estimators	300	100
	max_depth	None	None
	min_samples_leaf	1	1
	min_samples_split	2	2
	class_weight	None	None
**Light Gradient Boosting Machine**	n_estimators	100	300
	max_depth	10	10
	learning_rate	0.05	0.1
	num_leaves	31	50
**Gradient Boosting Classifier**	n_estimators	100	300
	max_depth	5	3
	learning_rate	0.2	0.2
	min_samples_leaf	1	1
	min_samples_split	2	5
